# The High Yield Expansion and Megakaryocytic Differentiation of Human Umbilical Cord Blood CD133^+^ Cells

**Published:** 2011-09-23

**Authors:** Mahin Nikougoftar Zarif, Masoud Soleimani, Hassan Abolghasemi, Naser Amirizade, Saeid Abroun, Saeid Kaviani

**Affiliations:** 1. Department of Hematology, Faculty of Medical Science, Tarbiat Modares University, Tehran, Iran; 2. Iran Blood Transfusion Organization Research Center, Tehran, Iran; 3. Department of Medical Science, Baghiyatallah University, Tehran, Iran

**Keywords:** Expansion, Differentiation, CD133^+^, Megakaryocyte, Cord Blood

## Abstract

**Objective: **Despite of many benefits, umbilical cord blood (UCB) hematopoietic stem cell (HSC) transplantation is associated with low number of stem cells and slow engraftment; in particular of platelets. So, expanded HSCs and co-transfusion of megakaryocyte (MK) progenitor cells can shorten this period. In this study, we evaluated the cytokine conditions for maximum expansion and MK differentiation of CD133^+^ HSCs. **Materials and Methods: **In this experimental study, The CD133^+^ cells were separated from three cord blood samples by magnetic activated cell sorting (MACS) method, expanded in different cytokine combinations for a week and differentiated in thrombopoietin (TPO) for the second week. Differentiation was followed by the flow cytometry detection of CD41 and CD61 surface markers. Colony forming unit (CFU) assay and DNA analysis were done for colonogenic capacity and ploidy assay. **Results: ** CD133^+^ cells showed maximum expansion in the stem span medium with stem cell factor (SCF) + FMS-like tyrosine kinase 3-ligand (Flt3-L) + TPO but the maximum differentiation was seen when CD133^+^ cells were expanded in stem span medium with SCF + Interleukin 3 (IL-3) + TPO for the first and in TPO for the second week. Colony Forming Unit-MK (CFU-MK) was formed in three sizes of colonies in the mega-cult medium. In the DNA analysis; 25.2 ± 6.7% of the cells had more than 2n DNA mass. **Conclusion: **Distinct differences in the MK progenitor cell count were observed when the cells were cultured in stem span medium with TPO, SCF, IL-3 and then the TPO in the second week. Such strategy could be applied for optimization of CD133^+^ cells expansion followed by MK differentiation.

## Introduction

 Transplantation of allogenic and autologous hematopoietic stem cells (HSCs) is used widely for reconstituting the hematopoietic cells after high dose chemotherapy and radiotherapy and some hematologic diseases. In the past, the main source of the HSC, for transplant was the bone marrow (1). Then mobilized peripheral blood by granulocyte-colony stimulation factor (G-CSF) was used as an alternative source of stem cells. It is more convenient and follows by a quicker recovery of neutrophils and platelets (2), but sometimes poor mobilization happens (3).The use of cord blood transplantation in pediatric patients has been established in 2000(4,5). However, it has two major limitations: the HSCs may be sufficient for children, but not for adults, and there is a delayed engraftment, especially in the platelets’ (Plt) recovery. Multiple plt transfusion carries the risk of alloantibody formation and plt refractoriness(6). Also clinical trials have shown that recombinant thrombopoietin (TPO) stimulates the megakaryocyte (MK) formation in vivo, but it does not shorten its maturation time (7). So, co-transfusion of HSC and MK progenitor cells can shorten this period. There are several protocols regarding the influence of some cytokines and chemokines to expand and differentiate HSCs including: Interlukin-3 (IL-3), IL-6, IL-9, IL-11, interferon- γ (IFN-γ), FMS-like tyrosine kinase 3ligand (Flt3-L), stem cell factor (SCF), TPO, erythropoietin (EPO), stromal derived factor-1 (SDF-1), and macrophage inflammatory protein-1 (MIP-1)(8-11).In some studies, the use of TPO as a key cytokine for megakaryocytic differentiation showed low expansion and early apoptosis in ex vivo cultures(12-14), while addition of other cytokines improved MK expansion and differentiation. In the present study, the maximum potential of in vitro expansion of CD133^+^ umbilical cord blood (UCB) cells in the presence of Flt3-L, TPO, SCF and IL-3 for a week, and optimal differentiation of expanded CD133^+^ cells in the presence of TPO as an MK active cytokine was studied.

## Materials and Methods

### Collection of cord blood

In this experimental study, human UCB samples were collected from consenting woman who had normal full-term pregnancy without any complications and signed the testimonial form. This research also was confirmed by Tarbiat Modares University Ethic Group. Cord blood samples were collected in 20 ml CPDA bags and processed within 24 hours of collections.

### CD133^+^ cell separation

Mononuclear cells were separated from the UCB, using Ficoll Hypaque (density 1077 g/cm^3^, Pharmacia, Sweden) density centrifugation at 2500 rpm for 30 minutes at 25℃, and washed by phosphate buffer saline containing 5% bovin serum albumin (Stem Cell Technology,Canada). The CD133^+^ fraction was enriched with Magnetic Activated Cell Sorting (MACS) method (Miltenyi Biotec, Canada) according to the manufacturer's instructions. The procedure was performed twice to obtain higher purity of the selected CD133^+^cells. The efficiency of purification was verified by flow cytometry (Partec PAS III,Germany), counterstaining with a Monoclonal Antibody (MoAb) CD133-PE (Miltenyi,Canada) and MoAb CD34- FITC(Miltenyi,Canada) ,also MoAb CD41-PE and CD61-FITC (DAKO, Denmark) were used to confirm the negativity of the MK series.

### Cell culture, expansion and differentiation

The UCB CD133^+^ cells were cultured in serum free stem span (Stem Cell Technology,Canada) medium in the tissue culture flask T_25_ and maintained at 37℃ in a fully humidified atmosphere with 5%CO_2_. Ten cytokine combinations were added at 1st and 3th day: 1- SCF (100 ng/ml) +TPO (100 ng/ml). 2- SCF (100 ng/ml) + TPO (100ng/ml) + Flt3-L (100 ng/ ml). 3- SCF (100 ng/ml) + TPO (100 ng/ml) + IL-3 (10 ng/ml). In order to MK differentiation, after a week ,the cells were counted and transferred into 6-well tissue-culture plates in serum free stem span media with TPO (100 ng/ml). The TPO was added twice a week. Differentiation was followed by Flow cytometric analysis of CD41 and CD61 surface marker expression. CFU-MK for colonogenic capacity and DNA analysis for ploidy detection of MK progenitors were done. 

### Colony forming unit-megakaryocyte

To evaluate the colonogenic capacity of MK differentiated cells, we used Mega-cult medium that is formulated to allow optimal detection of MK progenitors (Stem Cell Technologies, Canada). 100 µl of MK differentiated cells (confirmed by flow cytometry and suspended at 2×10^4^ cells per ml) were added to 2.0 ml of Mega-cult media (Stem Cell Technologies, Canada) and 1.2 ml of cold collagen solution, then were mixed and transferred into two 35mm petri dishes, which were in turn placed in a 100 mm petro dish along with an open 35 mm petri dish containing 3 ml sterile water to maintain optimal humidity. The petri dish was transferred into a 37℃ incubator with 5% CO_2_ and >95% humidity. After 14 days, the colonies were counted.

### Ploidy analysis

To evaluate the maturation stage of MK differentiated cells, DNA ploidy was measured by flow cytometry. For this purpose, the cells were incubated for 45 minutes at 37℃ with 0.1% Triton X-100 (Sigma,USA), RNAse (Sigma,USA) and propidium Iodide (Sigma,USA) to stain the DNA.

## Results

### CD133^+^ cells expansion

**Fig 1:**
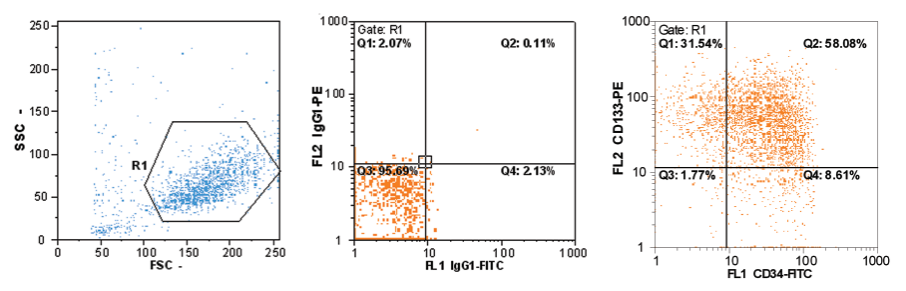
Purity of CD133^+^ cells. Left graph: Distribution of size and granulity, middle graph: Control isotype, Right graph: CD133^+^ versus CD34^+^ cells distribution.

 CD133^+^ cells were separated by MACS. The mean ± SD of total cells was 8.4 ± 2.8×105. Then, they were cultured in three conditions, and after 7 days of expansion, total count was done (Table 1) and purity of the CD133^+^ cells was detected by flow cytometry (Fig 1).

**Table 1:** Total number of cells, number of CD133^+^ cells and fold expansion of CD133^+^ cells during 7 days of culture (mean±SD)


Cytokine conditions	Day 0 Total cell density(×10_5_cells/flask T_25_	Day 7 Total cell density(×10_5_cells/flask T_25_	Day 7 Content of CD133^+^cells(%)	Day 7 Expansion fold of CD133^+^ cells	
SCF+TPO	8.4 ± 2.8	154.2 ± 84.0	66.8 ± 12.7	14.7 ± 7.5	
SCF+Flt3L+TPO	8.4 ± 2.8	570.0 ± 155.8	77.8 ± 5.8	62.1 ± 13.7	
SCF+IL-3+TPO	8.4 ± 2.8	442.9 ± 123.0	76.5 ± 4.4	46.9 ± 7.1	


**Table 2:** The percent of CD41^+^,CD61^+^and dual CD41^+^/CD61^+^cells and total number of CD41^+^ cells as megakaryocyte markers after 2 weeks culture in different expansion conditions(mean ± SD)


Cytokine conditions		CD41^+^(%)	CD61^+^(%)	CD41^+^/CD61^+^	CD41^+^number	
First week	Second week					
SCF+TPO	TPO	36.1 ± 5.4	12.9 ± 5.6	11.1 ± 4.1	18.4 ± 10.3×10^5^	
SCF+Flt3L+TPO	TPO	35.6 ± 10.8	15.8 ± 6.1	15.6 ± 5.4	78.6 ± 31.0×10^5^	
SCF+IL-3+TPO	TPO	90.9 ± 8.1	74.6 ± 7.9	74.0 ± 7.9	153.5 ± 44.3×10^5^	


### Cells differentiation

**Fig 2:**
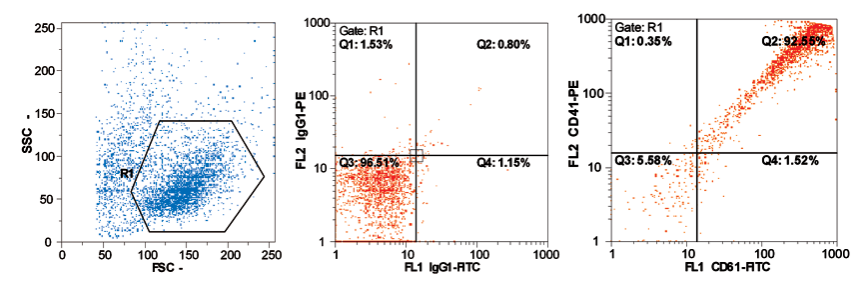
Percent of CD41+ and CD61+ cells. Left graph: Distribution of cell size and granulity, middle graph: Control isotype, Right graph: CD41+ versus CD61+ cells distribution. Bright expression of CD41+ and CD61+ cells should be considered.

Cell differentiation was evaluated by flow cytometry after two weeks of culture (Table 2, Fig 2). The most MK differentiated cells were formed in the third condition.

### CFU-MK

**Fig 3:**
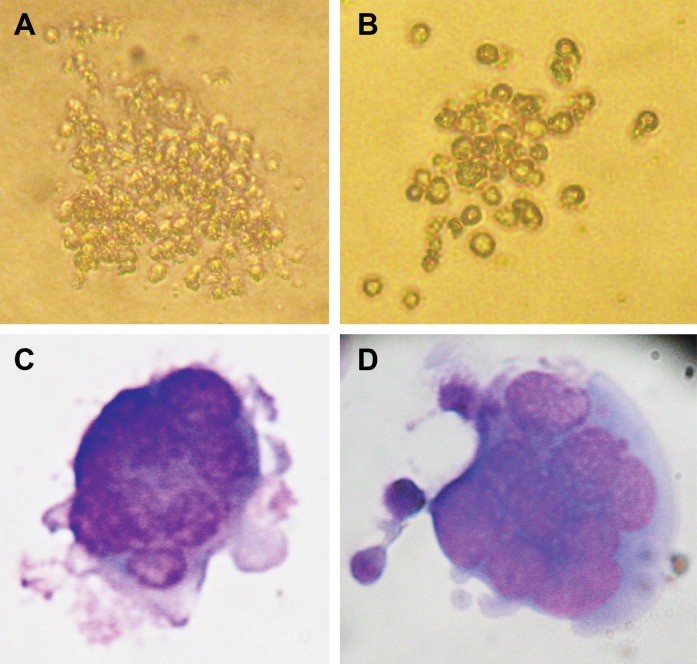
A. A large size and B. A medium size MK colony that formed in Mega-cult medium. C, D. Wright staining of MK .

CFU- colony formingunit-megakaryocyte produced three size colonies in the Mega-cult medium. There were 65.3 ± 13.5 small size colonies with 3-21 cells, 4.6 ± 1.5 medium size colonies with 21-49 cells and 2.3 ± 1.5 large size colonies with more than 49 cells (Fig 3).

### DNA analysis

**Fig 4:**
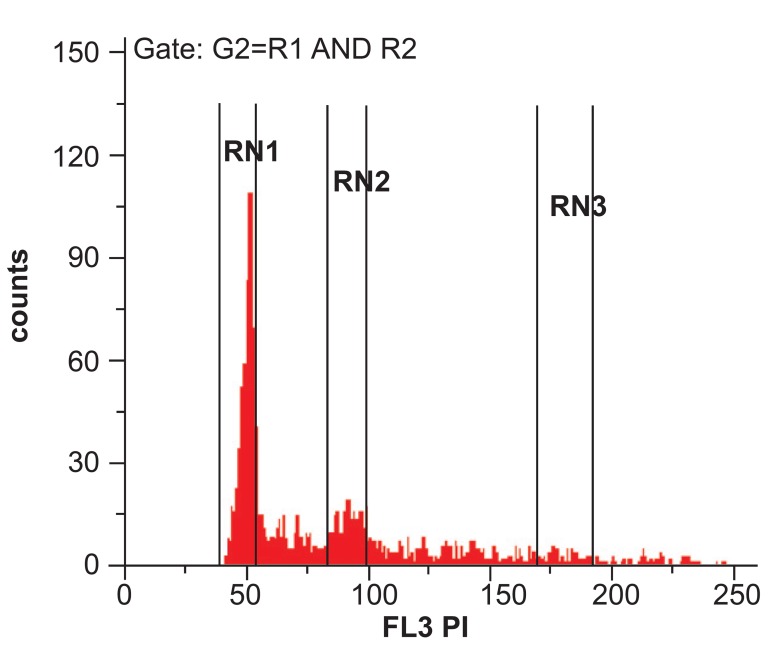
DNA analysis of a sample with more than 70% CD41^+^ cells. RN1 (G0G1 cells), RN2 (G2M cells), RN3 (4N Cells)

 The cells percent (mean ± SD) in the G0G1 phase was 45.9 ± 8.3 and in the 2N was 13.7 ± 3.5; this value for the cells in the 4N was 4.9 ± 1.3 and in total 25.2 ± 6.7% of the cells had more than 2N DNA mass (Fig 4).

### Discussion

UCB stem cells transplantation, despite of many benefits is associated with slow engraftment, in particular of platelets because of low numbers of HSC and MK progenitor cells. Possible approaches to expansion have been obtained, and co-transfusion of large number of ex vivo generated human MK cells is a way to shorten thrombocytopenia period.

In this study, we investigated the effects of various cytokine combinations in the high expansion and MK differentiation of CD133^+^ HSCs. We have used two -step cytokine conditions including: 1. Expansion with different cytokine cocktails and 2.differentiation with TPO. According to the results, though the expansion of CD133^+^ cells in SCF, TPO and Flt3-L cocktail is more than in other conditions and this is favorable with some studies (15-17), however, when the MK differentiation is the main aim of expansion, TPO, SCF and IL-3 are the best choice. IL-3 made the expanded cells more capable to differentiation (18). So the total amount of MK progenitors after two weeks of treatment was higher when we used TPO, SCF, IL-3 for CD133^+^ expansion. This finding is in agreement with Kashiwakura et al. (19)report that has provided the role of TPO, SCF, and IL-3 in CFU-Meg formation. Besides Piacibellond et al.(20) and Ueda et al.(21)reported that the combination of SCF, Flt3-L and TPO can expand more primitive HSCs and has lower effect on the progenitor cells. According to Kuter and Begly report, Flt3-L is dispensable(22),and TPO alone can induce high MK purity, but low MK expansion, which is the same as when TPO is combined with Flt3-L or IL-11(22, 23). In this manner, there are many studies on the other varieties of cytokines (IL-6, IL-9, IL-11, and some hormones) to identify optimum cocktails for expansion and differentiation(24, 25).For instance, Amiphosine can expand and produce MK progenitors as high as 83 folds(26).

 All together, we preferred to use one-week expansion prior to MK differentiation and used three group cytokine combinations, because of previous reports on their effect on MK expansion and purity differentiation.(27)

 For MK differentiation follow up, we used CD41 and CD61 surface expression detection. CD41 expresses earlier than other markers (28), and at the end of the second week, the bright expression of CD41 and CD61, as shown in (Fig 1), was observed.

The degree of MK progenitor maturation was tested by ploidy analysis in day the 14, and in average, about 25% of total cells had more than 2N ploidy, where most of the differentiated cells were megakaryoblast at that time. Further, based on previous studies, more ploidy of MKs could be obtained if the culture was followed more(29).

 Colonogenic capacity of MK progenitors is an important criterion. We used Mega-cult media designed for MK colonies growth. After 14 days, from the total of 1000 cells cultured in a 30 mm petri , about 70 colonies were formed. Because of limited Mega-cult culture data in other studies, comparison of our results with those of other achieved studies is not possible.

### Conclusion

We observed distinct differences in the MK progenitor cells count, when we used TPO, SCF, IL-3 and then TPO in the second week. Such strategy could be applied for optimization of CD133^+^ cells expansion followed by MK differentiation.
